# Effects of Diisononyl Phthalate on Atopic Dermatitis *in Vivo* and Immunologic Responses *in Vitro*

**DOI:** 10.1289/ehp.0901255

**Published:** 2009-11-19

**Authors:** Eiko Koike, Rie Yanagisawa, Kaori Sadakane, Ken-ichiro Inoue, Takamichi Ichinose, Hirohisa Takano

**Affiliations:** 1 Environmental Health Sciences Division, National Institute for Environmental Studies, Tsukuba, Japan; 2 Department of Health Sciences, Oita University of Nursing and Health Sciences, Oita, Japan

**Keywords:** antigen-presenting activity, atopic dermatitis, bone-marrow–derived dendritic cells, chemokines, diisononyl phthalate, eosinophils, mast cells, splenocytes

## Abstract

**Background:**

Diisononyl phthalate (DINP), a principal plasticizer in many polyvinyl chloride products, has been shown to have an adjuvant effect on immunoglobulin (Ig) production in mice. However, the effects of DINP on allergic diseases have not been fully elucidated.

**Objectives:**

In the present study we investigated the effects of DINP on atopic dermatitis (AD)-like skin lesions induced by *Dermatophagoides pteronyssinus* (Dp) in atopic-prone NC/Nga mice.

**Methods:**

Mice were injected intradermally with Dp on their ears and were exposed to DINP (0, 0.15, 1.5, 15, or 150 mg/kg/day) intraperitoneally. We evaluated clinical scores, ear thickening, histologic findings, protein expression of cytokines/chemokines in the ear, and serum levels of Ig and histamine. Furthermore, we investigated the effects of DINP on bone-marrow–derived dendritic cells (BMDCs) or splenocytes *in vitro*. After exposure to DINP (0–100 μM), cells were evaluated for phenotype and function.

**Results:**

DINP aggravated AD-like skin lesions related to Dp. The aggravation was consistent with eosinophilic inflammation, mast cell degranulation, and thymic stromal lymphopoietin (TSLP) expression in the ear. DINP enhanced the expression of cell surface activation markers on BMDCs and their production of TARC/CCL17 (thymus- and activation-regulated chemokine) and MDC/CCL22 (macrophage-derived chemokine), as well as their capacity to stimulate Dp-specific T-cell proliferation. DINP also enhanced interleukin-4 production and Dp-stimulated proliferation of splenocytes.

**Conclusions:**

DINP can aggravate AD-like skin lesions related to Dp. The mechanisms of the aggravation might be mediated, at least partly, through the TSLP-related activation of dendritic cells and by direct or indirect activation of the immune cells.

Phthalate esters, ubiquitously used as plasticizers in many polyvinyl chloride (PVC) products, have become widespread in the environment (Peijnenburg et al. 2008). Diisononyl phthalate (DINP) is used in flooring, wire and cable, dip coating, coated fabrics, tubing, shoes, sealants, and artificial leather, and humans may be exposed to DINP by the oral, dermal, and inhalation routes (Kavlock et al. 2002). It has been expected that general population exposure to DINP would not exceed levels of di-(2-ethylhexyl)phthalate (DEHP) (Kavlock et al. 2002), which are estimated at 3–30 μg/kg body weight/day (Doull et al. 1999). Plasticizers, including DINP, are not covalently bound to the plastics and can migrate into saliva and be swallowed (Earls et al. 2003; Kavlock et al. 2002). Thus, children may be exposed to higher levels of DINP than are adults because infants and small children mouth toys and other articles containing DINP (Babich et al. 2004; Kavlock et al. 2002).

Several epidemiologic studies have suggested that exposure to phthalate esters may be associated with development of asthma, wheezing, and allergic symptoms (Bornehag et al. 2004; Jaakkola et al. 1999, 2004, 2006). Bornehag et al. (2004) revealed the positive association between allergic asthma in children and phthalate esters in house dust. Thus, it is possible that phthalate esters in the environment may also be associated with development of the other allergic diseases such as atopic dermatitis (AD).

In our previous studies, DEHP enhanced AD-like skin lesions in atopic-prone NC/Nga mice at hundreds-fold lower levels than the no observed adverse effect level (NOAEL) determined from histologic changes in the liver of rodents (Takano et al. 2006; Yanagisawa et al. 2008). The enhancing effects of DEHP paralleled the infiltration of eosinophils, mast cell degranulation, and the expression of proinflammatory proteins in inflamed skin. Furthermore, we have shown that DEHP enhanced differentiation of bone-marrow–derived dendritic cells (BMDCs) and promoted T-helper 2 (T_H_2) cell response in splenocytes from NC/Nga mice *in vitro* (Koike et al. 2009).

The chronic health effects of DINP, including organ toxicity, carcinogenicity, and reproductive toxicity, have been reviewed in dietary studies (Babich et al. 2004; Kavlock et al. 2002). DINP, as a phthalate plasticizer with specific stereochemical and physicochemical characteristics, has also been shown to have an adjuvant effect on T_H_2-dependent immunoglobulin (Ig) production in mice (Larsen et al. 2002; Larsen and Nielsen 2008). However, the effects of DINP on allergic diseases including AD have remained unclear.

In the present study, we investigated the effects of DINP on AD-like skin lesions in atopic-prone NC/Nga mice *in vivo* and on the immunologic responses of BMDCs and splenocytes *in vitro*.

## Materials and Methods

### Animals

Six-week-old SPF NC/NgaTndCrlj male mice were purchased from Charles River Japan (Osaka, Japan) and used at 7 weeks of age (body weight, 20–23 g) and 11–15 weeks (body weight, 24–27 g) for *in vivo* and *in vitro* study, respectively. Mice were given sterile distilled water and a commercial diet (CE-2; CLEA Japan Inc., Tokyo, Japan) *ad libitum*, and were housed in an animal facility that was maintained at 22–26°C with 40–69% humidity and a 12/12-hr light/dark cycle under conventional conditions. The procedures for all animal studies were approved by the Institutional Review Board of Japan’s National Institute for Environmental Studies. Animals were treated humanely and with regard for alleviation of suffering.

### Protocol for *in vivo* study

Mice were divided into six groups and were injected intradermally on the ventral side of their right ears with saline or 5 μg mite extract [*Dermatophagoides pteronyssinus* (Dp); Cosmo Bio LSL, Tokyo, Japan] dissolved in 10 μL saline on study days 0, 3, 5, 8, 10, 12, 15, and 17 under anesthesia with 4% halothane (Takeda Pharmaceutical Company, Ltd., Osaka, Japan). DINP (Wako Pure Chemical Industries, Osaka, Japan), at a dose of 0, 0.15, 1.5, 15, or 150 mg/kg/day dissolved in 0.1 mL olive oil (vehicle), was injected intraperitoneally (IP) on days –5, 2, 9, and 16 from the first Dp treatment. Twenty-four hours after each Dp injection, we evaluated ear thickness and clinical scores as described previously (Takano et al. 2006). Twenty-four hours after the last injection of Dp (day 18), the animals were sacrificed, and histologic findings, protein levels of cytokines and chemokines in the ear tissue supernatants, and the levels of Ig and histamine in serum were evaluated.

### Histologic evaluation

Right ears of mice were removed 24 hr after the last Dp injection (day 18) and were fixed in 10% neutral phosphate-buffered formalin (pH 7.2) and embedded in paraffin. Sections (3 μm) were routinely stained with hematoxylin and eosin (H&E) or with toluidine blue (pH 4.0). Histologic analysis was performed using an AX80 microscope (Olympus, Tokyo, Japan). We measured the length of the cartilage and the numbers of infiltrated eosinophils and mast cells in each sample using a video micrometer (VM-30; Olympus). We also evaluated the degranulation of mast cells as nondegranulated (0%), mildly degranulated (0–50%), or severely degranulated (> 50%), as described previously (Takano et al. 2006).

### Quantitation of cytokines/chemokines in the ear tissue

Right ears of mice were removed 24 hr after the last injection of Dp (day 18) and were homogenized and centrifuged as previously described (Takano et al. 1997). Levels of interferon (IFN)-γ (Endogen, Cambridge, MA, USA), interleukin (IL)-4 (Amersham, Buckinghamshire, UK), IL-5 (Endogen), IL-13 (R&D Systems, Minneapolis, MN, USA), eotaxin (R&D Systems), eotaxin-2 (R&D Systems), and thymic stromal lymphopoietin (TSLP; R&D Systems) in the ear tissue supernatants were measured by enzyme-linked immunosorbent assay (ELISA) according to the manufacturers’ instructions. The detection limits of IFN-γ, IL-4, IL-5, IL-13, eotaxin, and TSLP were less than 10, 5, 5, 1.5, 3, and 2.63 pg/mL, respectively. The detection limit of eotaxin-2 was not defined, and the assay range was 15.6–1,000 pg/mL. The total protein level in the ear tissue supernatants was measured by the Bradford method using a protein assay kit (Bio-Rad, Hercules, CA, USA). The values of cytokines/chemokines were compensated with the total protein and were expressed as picograms per milligram of total protein.

### Quantitation of Ig and histamine in serum

Blood was sampled by cardiac puncture 24 hr after the last injection of Dp (day 18) and serum was collected. Levels of Dp-specific IgG_1_ were measured by ELISA with solid-phase antigen, as previously described (Sadakane et al. 2002). Levels of total IgE antibodies and histamine in serum were measured by OptELISA Set Mouse IgE (BD Biosciences, San Diego, CA, USA) and Histamine Enzyme Immunoassay Kit (SPI-BIO, Montigny le Bretonneux, France), respectively, according to the manufacturers’ instructions.

### Cell preparation for *in vitro* study

For the *in vitro* study, bone marrow cells and splenocytes were prepared as previously described (Koike et al. 2009). Briefly, the marrow was flushed with Dulbecco’s calcium- and magnesium-free, phosphate-buffered saline (PBS; Takara Bio Inc., Shiga, Japan) and passed through nylon mesh; the red blood cells were then lysed with ammonium chloride. The spleen was pushed through a sterile stainless-steel wire mesh, and the red blood cells were similarly lysed. The cells were centrifuged at 400 × *g* for 5 min at 20°C. After washing with PBS, the cells were resuspended in culture medium R10, consisting of GIBCO RPMI 1640 medium (Invitrogen, Grand Island, NY, USA) supplemented with 10% heat-inactivated fetal bovine serum (MP Biomedicals Inc., Eschwege, Germany), 100 U/mL penicillin and 100 μg/mL streptomycin (Sigma, St. Louis, MO, USA), and 50 μM 2-mercaptoethanol (Invitrogen). The numbers of viable cells were determined by the trypan blue (Invitrogen) exclusion method.

### Differentiation of BMDCs

Bone marrow cells (4 × 10^5^/mL) were cultured in R10 medium containing 20 ng/mL recombinant mouse granulocyte–macrophage colony-stimulating factor (GM-CSF; Sigma) at 37°C in a 5% CO_2_/95% air atmosphere. On day 3, the same volume of medium was added to the culture, and on day 6, half the medium was replaced with fresh medium. On day 8, nonadherent and loosely adherent cells were collected by gentle pipetting. The differentiated BMDCs were centrifuged at 400 × *g* for 5 min at 20°C and resuspended in fresh medium. The numbers of viable cells were determined by the trypan blue exclusion method.

### Exposure of immune cells to DINP

DINP was dissolved in dimethyl sulfoxide (DMSO; Sigma) and diluted with R10. The DINP solution was sonicated for 3 min using an ultrasonic disrupter (UD-201; TOMY, Tokyo, Japan). In the presence of GM-CSF (10 ng/mL), BMDCs (1 × 10^6^/mL) were exposed to DINP (0, 0.1, 1, 10, or 100 μM) in R10 containing 0.1% DMSO for 24 hr. Thereafter, their chemokine production, phenotypes, and antigen-presenting activity were evaluated. Next, splenocytes (1 × 10^6^/mL) were exposed to DINP (0, 0.1, 1, 10, or 100 μM) in R10 containing 0.1% DMSO for 24 hr, and their cytokine production was examined. Moreover, splenocytes (1 × 10^6^/mL) were exposed to DINP (0, 0.001, 0.01, 0.1, 1, or 10 μM) in R10 containing 0.1% DMSO in the presence of Dp (10 μg/mL) for 72 hr. Thereafter, antigen-stimulated proliferation and cytokine production of the cells were measured. Each experiment was performed using three individual cultures obtained from three animals, and two or three independent experiments were repeated.

### Fluorescence-activated cell-sorting (FACS) analysis

For FACS analysis of BMDCs, we used the following monoclonal antibodies: major histocompatibility complex (MHC) class II I-A/I-E (2G9, rat IgG_2a_ κ, fluorescein isothiocyanate conjugated; BD Biosciences, San Jose, CA, USA); co-stimulatory molecules CD80 [16-10A1, Ar Ham IgG_1_ κ, phycoerythrin (PE) conjugated; BD Biosciences] and CD86 (GL1, rat IgG_2a_ κ, PE conjugated; BD Biosciences); and chemokine receptors CCR7 (4B12, rat IgG_2a_ κ, PE conjugated; Biolegend, San Diego, CA, USA) and CXCR4 (2B11/CXCR4, rat IgG_2b_ κ, PE conjugated; BD Biosciences). After DINP exposure, the cells (3–5 × 10^5^) were resuspended in 100 μL PBS with 0.3% bovine serum albumin and 0.05% sodium azide (Wako Pure Chemical Industries) and were incubated with 1 μg of each antibody for 30 min on ice. After incubation, the cells were washed, and the fluorescence was measured by a FACSCalibur (BD, Franklin Lakes, NJ, USA). For each sample, we collected fluorescence data from 10,000 cells and positive cells were expressed as percent of total events.

### Antigen-presenting activity

We evaluated BMDC function by antigen-presenting activity and stimulating capacity for cytokine production from responder T cells as previously described (Koike et al. 2009). Briefly, DINP-exposed BMDCs were treated with 50 μg/mL mitomycin C (Kyowa Hakko Kogyo, Tokyo, Japan) for 20 min at 37°C. Splenocytes were prepared from NC/Nga mice sensitized with 50 μg Dp and 1 mg aluminium hydroxide (three mice per experiment). T cells were isolated from the splenocytes using a nylon fiber column (Wako Pure Chemical Industries). Thereafter, Dp-sensitized T cells (2 × 10^5^) and BMDCs (5 × 10^3^) were cocultured in the presence of 2 μg Dp in 200 μL R10 medium in 96-well flat-bottom plates. The cocultures were performed in triplicate at 37°C in a 5% CO_2_/95% air atmosphere. After 91 hr, we measured T-cell proliferation as an indicator of antigen-presenting activity, and cytokine production from T cells.

### Cell proliferation assay

We measured cell proliferation using a Cell-Proliferation ELISA kit (Roche Molecular Biochemicals, Mannheim, Germany) according to the manufacturer’s instructions. This technique is based on the incorporation of the pyrimidine analogue 5-bromo-2′-deoxyuridine (BrdU) instead of thymidine into the DNA of proliferating cells. Cell proliferation was measured by adding BrdU to each well 20 hr before the measurement.

### Quantitation of cytokines/chemokines in the culture supernatants

The levels of thymus- and activation-regulated chemokine (TARC/CCL17; R&D Systems), macrophage-derived chemokine (MDC/CCL22; R&D Systems), and IL-12p40 (Endogen) in the BMDC culture supernatants and the levels of IFN-γ (Endogen), IL-4 (Amersham), IL-10 (Endogen), and IL-17 (R&D Systems) in the supernatants of BMDCs and T-cell cocultures or splenocyte cultures were measured by ELISA according to the manufacturers’ instructions. The detection limits of TARC/CCL17, MDC/CCL22, IL-12p40, IFN-γ, IL-4, IL-10, and IL-17 were less than 5, 1.2, 3, 10, 5, 12, and 5 pg/mL, respectively.

### Statistical analysis

Data are presented as mean ± SE. The significance of variation among different groups was determined by one-way analysis of variance or Kruskal-Wallis analysis. Differences among groups were analyzed using Dunnett’s or Steel multiple comparison test (Excel Statistics 2006, Social Survey Research Information Co., Ltd., Tokyo, Japan). A *p*-value < 0.05 is considered statistically significant.

## Results

### Effects of DINP on AD-like skin lesions

**Figure 1:**
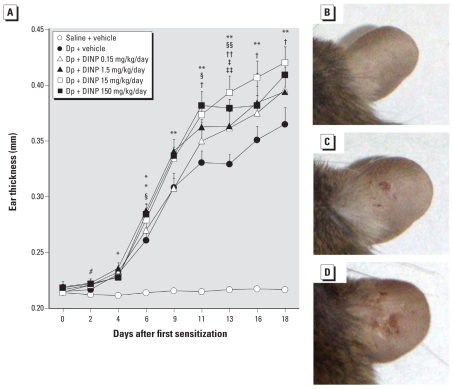
Effects of DINP on AD-like skin lesions induced by Dp on mouse ears. (*A*) Thickness of the ears 24 hr after each intradermal injection of Dp (mean ± SE of 12 animals per group). (*B*–*D*) Macroscopic features of the ears from the saline + vehicle group (*B*), Dp + vehicle group (*C*), and Dp + DINP 15 mg/kg/day group (*D*) 24 hr after the last injection of Dp. *p < 0.05, and **p < 0.01 for Dp-treated groups compared with the saline + vehicle group. ^#^*p* < 0.05 for Dp + DINP groups compared with the saline + vehicle group. ^§^*p* < 0.05, and ^§§^*p* < 0.01 for Dp + DINP 150 mg/kg/day versus Dp + vehicle. ^†^*p* < 0.05, and ^††^*p* < 0.01 for Dp + DINP 15 mg/kg/day versus Dp + vehicle. ^‡^*p* < 0.05 for Dp + DINP 1.5 mg/kg/day versus Dp + vehicle. ^‡‡^*p* < 0.05 for Dp + DINP 0.15 mg/kg/day versus Dp + vehicle.

To evaluate whether DINP affects AD-like skin lesions induced by Dp, we examined ear thickening and observed macroscopic features. Intradermal injection of Dp significantly enhanced ear thickening compared with saline injection from 4 days after the first injection of Dp (Figure 1A; *p* < 0.05). IP injection with DINP significantly enhanced ear thickening compared with vehicle in the presence of intradermal Dp from 6 days after the first injection of Dp (*p* < 0.05). However, we observed no dose-dependent effects of DINP. The ear thickening was most prominent in animals treated with 15 mg/kg/day DINP in the presence of intradermal Dp (*p* < 0.05). This result paralleled the clinical scores, including dryness, wound severity, and edema (data not shown). We observed macroscopic features demonstrating that DINP aggravated AD-like skin lesions related to Dp (Figure 1B–D).

### Histologic changes in the skin

**Figure 2:**
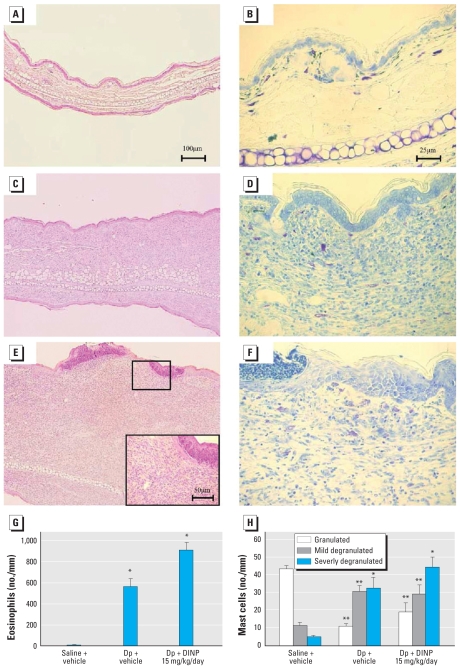
Effects of DINP on histologic changes in the ear 24 hr after the last intradermal injection of Dp. Photomicrographs of ear tissue sections from the saline + vehicle group (*A*, *B*), Dp + vehicle group (*C*, *D*), and Dp + DINP 15 mg/kg/day group (*E*, *F*) stained with H&E (*A*, *C*, *E*) or toluidine blue (*B*, *D*, *F*) and observed at 100× (*A*, *C*, *E*) and 400× (*B*, *D*, *F,* and *E* inset); data are representative of five animals per group. The infiltration of eosinophils (*G*) and the degranulation of mast cells (*H*) evaluated as the number of cells (mean ± SE) per millimeter of cartilage from five animals per group. *p < 0.05, and **p < 0.01 for Dp-treated groups compared with the saline + vehicle group.

To evaluate whether DINP affects histologic changes in the skin related to Dp, we examined the skin specimens stained with H&E (Figure 2A,C,E) or toluidine blue (Figure 2B,D,F). We found no pathologic changes in the saline plus vehicle group (Figure 2A,B). Intradermal injection of Dp induced the infiltration of eosinophils into the skin lesions compared with saline injection (Figure 2A,C,G; *p* < 0.05). IP injection with 15 mg/kg/day DINP tended to aggravate the infiltration of eosinophils into the skin lesion compared with vehicle in the presence of intradermal Dp (Figure 2C,E,G). In overall trend, these changes paralleled the severity of mast cell degranulation (Figure 2B,D,F,H).

### Protein expression of cytokines/chemokines in the skin

We investigated whether DINP affects the protein expression of T_H_1/T_H_2 cytokines and chemokines in the skin related to Dp. Intradermal injection of Dp significantly increased the expression of IL-4, IL-5, and IL-13 and significantly decreased the expression of IFN-γ compared with saline injection [see Supplemental Material, Table 1 (doi:10.1289/ehp.0901255); IL-4, *p* < 0.01; IL-5, *p* < 0.05; IL-13, *p* < 0.01; IFN-γ, *p* < 0.01]. IP treatment with DINP did not clearly affect the expression of these cytokines, although DINP tended to decrease IFN-γ expression compared with vehicle in the presence of intradermal Dp. Dp injection alone increased the expression of eotaxin, eotaxin-2, and TSLP compared with saline injection (see Supplemental Material, Table 1; eotaxin, *p* < 0.01). However, DINP significantly decreased the expression of eotaxin and eotaxin-2 compared with vehicle in the presence of intradermal Dp (*p* < 0.05). On the other hand, DINP at 0.15 mg/kg/day significantly increased TSLP expression compared with vehicle in the presence of intradermal Dp (*p* < 0.05). The expression of IL-12p40 was not detected (data not shown).

### Effects of DINP on the levels of Ig and histamine in serum

To evaluate the adjuvant capacity of DINP for Ig production, we measured the levels of Dp-specific IgG_1_ and total IgE in serum. We also investigated whether DINP affects histamine release in serum related to Dp. Intradermal injection of Dp significantly increased the levels of Dp-specific IgG_1_ and total IgE and tended to increase histamine levels in serum compared with saline injection [see Supplemental Material, Table 1 (doi:10.1289/ehp.0901255); *p* < 0.01]. IP DINP treatment did not affect the levels of Dp-specific IgG_1_ and total IgE in serum. In the presence of intradermal Dp, DINP significantly increased histamine levels in serum compared with saline injection (*p* < 0.01), except at 1.5 mg/kg/day. However, we found no significant difference between DINP and vehicle in the presence of intradermal Dp.

### Effects of DINP on BMDCs *in vitro*

**Figure 3:**
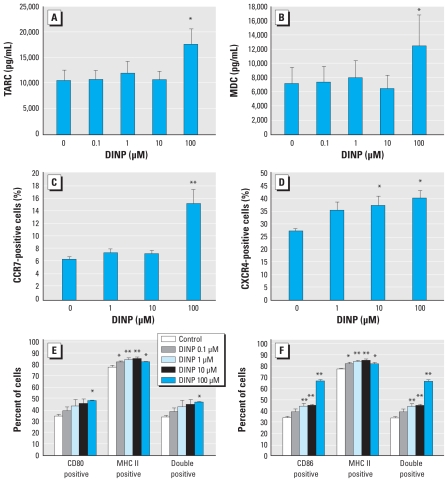
Effects of DINP on the production of chemokines and the expression of cell surface molecules on BMDCs. Levels of TARC/CCL17 (*A*) and MDC/CCL22 (*B*) in the BMDC culture supernatant after DINP exposure for 24 hr as measured by ELISA. (*C,D*) Expression of chemokine receptors CCR7 (*C*) and CXCR4 (*D*), and (*E,F*) expression of cell surface molecules associated with antigen presentation, MHC class II (MHC II) and CD80 (*E*) or MHC II and CD86 (*F*), on BMDCs after 24-hr DINP exposure as analyzed by flow cytometry. Data are the mean ± SE of three individual cultures from three animals and are representative of two or three independent experiments. *p < 0.05, and **p < 0.01 compared with control.

**Figure 4:**
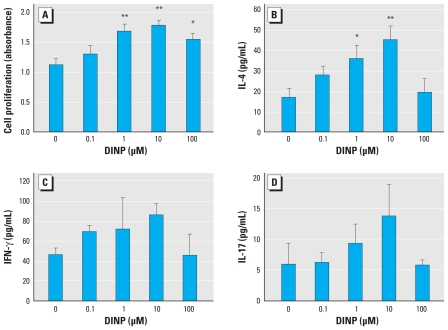
Effects of DINP on antigen-presenting activity of BMDCs. (*A*) Dp-specific antigen-presenting activity of BMDCs exposed to DINP for 24 hr evaluated by BrdU incorporation into proliferating T cells. Levels of IL-4 (*B*), IFN-γ (*C*), and IL-17 (*D*) in the cocultures of T cells and BMDCs exposed to DINP measured by ELISA. Data are the mean ± SE of three individual cultures from three animals and are representative of three independent experiments. *p < 0.05, and **p < 0.01 compared with control.

To evaluate whether DINP affects the phenotypes and function of BMDCs in NC/Nga mice, we examined the production of T_H_1/T_H_2 cytokines and chemokines, the expression of cell surface molecules, and antigen-presenting activity of BMDCs after exposure to DINP *in vitro*. DINP exposure for 24 hr significantly increased the production of T_H_2 chemokines, TARC/CCL17 (Figure 3A; 100 μM DINP, *p* < 0.05) and MDC/CCL22 (Figure 3B; 100 μM DINP, *p* < 0.05), from BMDCs compared with control (0 μM DINP). We did not detect the T_H_1 cytokine IL-12p40 in any BMDC cultures (data not shown). DINP also significantly increased the expression of the chemokine receptors CCR7 (Figure 3C; 100 μM DINP, *p* < 0.01) and CXCR4 (Figure 3D; 10 or 100 μM DINP, *p* < 0.05), MHC class II (Figure 3E,F; 1 or 10 μM DINP, *p* < 0.01; 0.1 or 100 μM DINP, *p* < 0.05), CD80 (Figure 3E; 100 μM DINP, *p* < 0.05), and CD86 (Figure 3F; 1, 10, or 100 μM DINP, *p* < 0.01) on BMDCs compared with controls. After the increases, Dp-specific antigen-presenting activity of BMDCs was significantly enhanced by DINP compared with control (Figure 4A; 1 or 10 μM DINP, *p* < 0.01; 100 μM DINP, *p* < 0.05). The levels of IL-4 in the cocultures of T cells and BMDCs exposed to DINP were also increased compared with controls (Figure 4B; 1 μM DINP, *p* < 0.05; 10 μM DINP, *p* < 0.01). The levels of IFN-γ (Figure 4C) and IL-17 (Figure 4D) tended to increase in response to DINP exposure in the same manner as IL-4 without significance. The level of IL-10 was lower than the detection limit (data not shown).

### Effects of DINP on splenocytes *in vitro*

**Figure 5:**
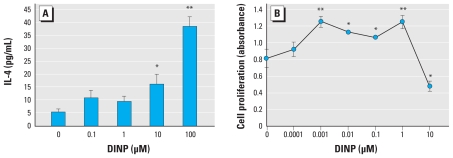
Effects of DINP on IL-4 production and antigen-stimulated proliferation of splenocytes. (*A*) Level of IL-4 in the splenocyte culture supernatant after exposure to DINP for 24 hr measured by ELISA. (*B*) Proliferation of splenocytes after exposure to DINP in the presence of Dp for 72 hr evaluated by BrdU incorporation into proliferating cells. Data are the mean ± SE of three individual cultures from three animals and are representative of three independent experiments. *p < 0.05, and **p < 0.01 compared with control.

To evaluate whether DINP affects the activation of splenocytes in NC/Nga mice, we examined the production of T_H_1/T_H_2 cytokines and Dp-stimulated proliferation of splenocytes after exposure to DINP *in vitro*. DINP exposure for 24 hr significantly increased IL-4 production from splenocytes compared with controls (Figure 5A; 10 μM DINP, *p* < 0.05; 100 μM DINP, *p* < 0.01). The levels of IL-10, IFN-γ, and IL-12p40 were < 10 pg/mL, and we observed no effects of DINP (data not shown). On the other hand, 72-hr exposure to DINP in the presence of Dp significantly increased proliferation of splenocytes at 0.001–1 μM and decreased the proliferation at 10 μM compared with controls (Figure 5B; 0.001 or 1 μM DINP, *p* < 0.01; 0.01, 0.1, or 10 μM DINP, *p* < 0.05).

## Discussion

In the present study, we found that DINP aggravated AD-like skin lesions related to Dp in NC/Nga mice. The aggravation was consistent with the degree of eosinophilic inflammation, mast cell degranulation, and TSLP expression in the inflamed ear. DINP also enhanced the expression of cell surface activation markers on BMDCs; the production of TARC/CCL17 and MDC/CCL22; and the Dp-specific antigen presentation, IL-4 production, and Dp-stimulated proliferation of splenocytes from NC/Nga mice *in vitro*.

DINP is ubiquitously used in PVC products and, along with DEHP, is widespread in the environment. Although previous studies have shown that DINP possesses adjuvant activity characterized by antigen-specific IgG_1_ antibody production (Larsen et al. 2002; Larsen and Nielsen 2008), the effects of DINP on allergic diseases or symptoms and on underlying cellular and molecular mechanisms remain unclear.

The present study showed that DINP (0.15, 1.5, 15, or 150 mg/kg/day) caused aggravation of AD-like skin lesions related to Dp in NC/Nga mice, as evidenced by macroscopic (Figure 1) and microscopic (Figure 2) examination. We observed no dose-dependent effects of DINP, and the symptoms were most prominent on exposure to 15 mg/kg/day DINP in the presence of intradermal Dp (Figure 1A). This dose of DINP has been reported as a NOAEL for general chronic oral toxicity in rats (Lington et al. 1997). Additionally, based on oral toxicologic studies on DINP in rats, the NOAEL for developmental toxicity and reproductive toxicity is 100–200 and 665–802 mg/kg/day, respectively (Kavlock et al. 2002). Accordingly, our present results indicate that DINP can aggravate AD-like skin lesions related to Dp at lower levels than those used in previous studies, whereas a NOAEL based on oral intake cannot be directly compared with an injected dose.

Compared with vehicle treatment, DINP exposure tended to increase histamine release in serum [see Supplemental Material, Table 1 (doi:10.1289/ehp.0901255)] in the presence of intradermal Dp. The increases in histamine may be one of the mechanisms underlying the aggravation of AD-like skin lesions by DINP. In contrast, DINP did not clearly affect Ig levels or the expression of T_H_1/T_H_2 cytokines such as IFN-γ, IL-4, IL-5, and IL-13. However, DINP significantly decreased eotaxin and eotaxin-2 expression in ear tissue in the presence of intradermal Dp, compared with vehicle (see Supplemental Material, Table 1), even though eosinophilic inflammation and mast cell degradation in the ear were present in parallel with the aggravation of AD-like skin lesions (Figure 2). This result differed from that in our previous study on DEHP (Takano et al. 2006), in which we observed increased expression of eotaxin and macrophage inflammatory protein-1α in the ear. It is possible that DINP induced the expression of T_H_2 cytokines and chemokines at an earlier phase and that this induction might contribute to the aggravation of the symptoms. A time-course study is needed to elucidate the correlation between symptoms and inflammatory mediators. In addition, it is possible that DINP may aggravate AD-like skin lesions through mechanisms different from those of DEHP. For instance, DINP enhanced TSLP expression compared with vehicle in the presence of intradermal Dp (see Supplemental Material, Table 1). Previous studies have shown that TSLP is associated with AD and asthma in humans (Soumelis et al. 2002; Ying et al. 2005) and mice (Li et al. 2005; Zhou et al. 2005). TSLP is an epithelial-cell–derived IL-7-like cytokine that strongly activates dendritic cells. TSLP-activated dendritic cells produce T_H_2-attracting chemokines (Soumelis et al. 2002) and then induce T_H_2 cell activation, initiating allergic inflammation by triggering IgE production, eosinophilia, and mucus production (Liu 2006; Liu and Frederick 2009). In a preliminary study, we observed that exposure to 0.15 mg/kg/day DINP further enhanced the expression of activation markers of antigen-presenting cells, including dendritic cells, and that these cell numbers in local lymph node cells compared with vehicle in the presence of intradermal Dp (data not shown). This result is consistent with the enhancement of TSLP expression. Therefore, DINP may activate dendritic cells in Dp-related inflammatory sites through the enhancement of TSLP production. The activated dendritic cells can migrate into local lymph nodes, leading to acceleration of antigen presentation and T_H_2 cell activation.

Thus, we focused on dendritic cells and investigated the effects of DINP on BMDCs *in vitro*. In the present study we found that 24-hr DINP exposure significantly increased the production of the T_H_2-attracting chemokines TARC/CCL17 (Figure 3A) and MDC/CCL22 (Figure 3B) but not the T_H_1-polarizing cytokine IL-12p40 (data not shown), and increased the expression of CCR7, CXCR4 (Figure 3C,D), MHC class II, CD80, and CD86 (Figure 3E,F) in BMDCs. CCR7 and CXCR4 are chemokine receptors for regulating dendritic cell function and migration into draining lymph nodes (Kabashima et al. 2007). MHC class II (Niederhuber and Shreffler 1977) and costimulatory molecules CD80 and CD86 (Freeman et al. 1993; Lenschow et al. 1993) are essential for antigen presentation. DINP exposure significantly enhanced Dp-specific antigen-presenting activity of BMDCs compared with the control (Figure 4A). DINP-exposed BMDCs consequently stimulated IL-4 production in the cocultures with Dp-sensitized T cells (Figure 4B). The production of IFN-γ and IL-17 in the cocultures was not significantly altered by DINP exposure (Figure 4C,D). DINP also enhanced BMDC differentiation after 6-day culture of bone marrow cells in the presence of DINP (data not shown). However, DINP more clearly affected BMDC activation instead of BMDC differentiation. In contrast, our previous study (Koike et al. 2009) showed that DEHP may predominantly affect BMDC differentiation rather than BMDC activation. The different immune stimulatory effects of these phthalates may be related to their chemical structures and properties. Larsen and Nielsen (2008) suggested that the adjuvant effects of phthalates are highly influenced by both stereochemical and physicochemical properties.

Second, we investigated the effects of DINP on splenocytes *in vitro* in order to evaluate whether DINP affects systemic immune responses. Exposure to DINP for 24 hr significantly increased IL-4 production from splenocytes in a concentration-dependent manner (Figure 5A). The levels of IL-10, IFN-γ, and IL-12p40 were < 10 pg/mL, and we observed no effects of DINP (data not shown). In a previous study Lee et al. (2004) showed that *in vitro* and/or *in vivo* exposure to DEHP and DINP enhances IL-4 production in CD4^+^ T cells and activation of IL-4 gene promoter containing binding sites for the NFAT (nuclear factor of activated T cells) transcription factor. That study and our present findings suggest that DEHP and DINP may enhance allergic responses by enhancing IL-4 production in CD4^+^ T cells via an NFAT-dependent pathway. Further studies are needed to identify the signaling pathways, including transcriptional factors, associated with the effects of DEHP and DINP on T cells. Moreover, exposure to DINP at low concentrations (0.001–1 μM) for 72 hr significantly increased Dp-stimulated splenocyte proliferation (Figure 5B). The DINP-induced increase showed maximal activity at these concentrations and then decreased. We previously showed a similar effect with DEHP (Koike et al. 2009). Therefore, these results suggest that prolonged exposure of splenocytes to low concentrations of phthalates such as DEHP and DINP can activate their proliferation in the presence of antigen.

## Conclusions

DINP can aggravate AD-like skin lesions related to Dp in NC/Nga mice at the same levels as the NOAEL for general toxicity. TSLP, in particular, may play a crucial role in this aggravation. Furthermore, DINP can enhance T_H_2 responses through the activation of BMDCs and splenocytes *in vitro*. The mechanisms underlying the aggravation of AD-like skin lesions by DINP may be linked, in part, to the enhanced TSLP-mediated activation of dendritic cells and a direct or indirect activation of the immune cells.

## References

[b1-ehp-118-472] Babich MA, Chen S-B, Greene MA, Kiss CT, Porter WK, Smith TP (2004). Risk assessment of oral exposure to diisononyl phthalate from children’s products. Regul Toxicol Pharmacol.

[b2-ehp-118-472] Bornehag CG, Sundell J, Weschler CJ, Sigsgaard T, Lundgren B, Hasselgren M (2004). The association between asthma and allergic symptoms in children and phthalates in house dust: a nested case–control study. Environ Health Perspect.

[b3-ehp-118-472] Doull J, Cattley R, Elcombe C, Lake BG, Swenberg J, Wilkinson C (1999). A cancer risk assessment of di(2-ethylhexyl)phthalate: application of the new US EPA risk assessment guidelines. Regul Toxicol Pharmacol.

[b4-ehp-118-472] Earls AO, Axford IP, Braybrook JH (2003). Gas chromatography-mass spectrometry determination of the migration of phthalate plasticisers from polyvinyl chloride toys and childcare articles. J Chromatogr A.

[b5-ehp-118-472] Freeman GJ, Borriello F, Hodes RJ, Reiser H, Gribben JG, Ng JW (1993). Murine B7-2, an alternative CTLA4 counter-receptor that costimulates T cell proliferation and interleukin 2 production. J Exp Med.

[b6-ehp-118-472] Jaakkola JJ, Ieromnimon A, Jaakkola MS (2006). Interior surface materials and asthma in adults: a population-based incident case-control study. Am J Epidemiol.

[b7-ehp-118-472] Jaakkola JJ, Oie L, Nafstad P, Botten G, Samuelsen SO, Magnus P (1999). Interior surface materials in the home and the development of bronchial obstruction in young children in Oslo, Norway. Am J Public Health.

[b8-ehp-118-472] Jaakkola JJ, Parise H, Kislitsin V, Lebedeva NI, Spengler JD (2004). Asthma, wheezing, and allergies in Russian schoolchildren in relation to new surface materials in the home. Am J Public Health.

[b9-ehp-118-472] Kabashima K, Shiraishi N, Sugita K, Mori T, Onoue A, Kobayashi M (2007). CXCL12-CXCR4 engagement is required for migration of cutaneous dendritic cells. Am J Pathol.

[b10-ehp-118-472] Kavlock R, Boekelheide K, Chapin R, Cunningham M, Faustman E, Foster P (2002). NTP Center for the Evaluation of Risks to Human Reproduction: phthalates expert panel report on the reproductive and developmental toxicity of di-isononyl phthalate. Reprod Toxicol.

[b11-ehp-118-472] Koike E, Inoue K-I, Yanagisawa R, Takano H (2009). Di-(2-ethylhexyl) phthalate affects immune cells from atopic prone mice in vitro. Toxicology.

[b12-ehp-118-472] Larsen ST, Lund RM, Nielsen GD, Thygesen P, Poulsen OM (2002). Adjuvant effect of di-*n*-butyl-, di-*n*-octyl-, di-iso-nonyl- and di-iso-decyl phthalate in a subcutaneous injection model using BALB/c mice. Pharmacol Toxicol.

[b13-ehp-118-472] Larsen ST, Nielsen GD (2008). Structure-activity relationship of immunostimulatory effects of phthalates. BMC Immunology.

[b14-ehp-118-472] Lee MH, Park J, Chung SW, Kang BY, Kim SH, Kim TS (2004). Enhancement of interleukin-4 production in activated CD4+ T cells by diphthalate plasticizers via increased NF-AT binding activity. Int Arch Allergy Immunol.

[b15-ehp-118-472] Lenschow DJ, Su GH, Zuckerman LA, Nabavi N, Jellis CL, Gray GS (1993). Expression and functional significance of an additional ligand for CTLA-4. Proc Natl Acad Sci USA.

[b16-ehp-118-472] Li M, Messaddeq N, Teletin M, Pasquali J-L, Metzger D, Chambon P (2005). Retinoid X receptor ablation in adult mouse keratinocytes generates an atopic dermatitis triggered by thymic stromal lymphopoietin. Proc Natl Acad Sci USA.

[b17-ehp-118-472] Lington AW, Bird MG, Plutnick RT, Stubblefield WA, Scala RA (1997). Chronic toxicity and carcinogenic evaluation of diisononyl phthalate in rats. Fundam Appl Toxicol.

[b18-ehp-118-472] Liu Y-J (2006). Thymic stromal lymphopoietin: master switch for allergic inflammation. J Exp Med.

[b19-ehp-118-472] Liu Y, Frederick WA (2009). TSLP in epithelial cell and dendritic cell cross talk. Adv Immunol.

[b20-ehp-118-472] Niederhuber JE, Shreffler DC (1977). Anti-Ia serum blocking of macrophage function in the in vitro humoral response. Transplant Proc.

[b21-ehp-118-472] Peijnenburg WJGM, Sven Erik J, Brian F (2008). Phthalates. Encyclopedia of Ecology.

[b22-ehp-118-472] Sadakane K, Takano H, Ichinose T, Yanagisawa R, Shibamoto T (2002). Formaldehyde enhances mite allergen-induced eosinophilic inflammation in the murine airway. J Environ Pathol Toxicol Oncol.

[b23-ehp-118-472] Soumelis V, Reche PA, Kanzler H, Yuan W, Edward G, Homey B (2002). Human epithelial cells trigger dendritic cell mediated allergic inflammation by producing TSLP. Nat Immunol.

[b24-ehp-118-472] Takano H, Yanagisawa R, Inoue K, Ichinose T, Sadakane K, Yoshikawa T (2006). Di-(2-ethylhexyl) phthalate enhances atopic dermatitis-like skin lesions in mice. Environ Health Perspect.

[b25-ehp-118-472] Takano H, Yoshikawa T, Ichinose T, Miyabara Y, Imaoka K, Sagai M (1997). Diesel exhaust particles enhance antigen-induced airway inflammation and local cytokine expression in mice. Am J Respir Crit Care Med.

[b26-ehp-118-472] Yanagisawa R, Takano H, Inoue K, Koike E, Sadakane K, Ichinose T (2008). Effects of maternal exposure to di-(2-ethylhexyl) phthalate during fetal and/or neonatal periods on atopic dermatitis in male offspring. Environ Health Perspect.

[b27-ehp-118-472] Ying S, O’Connor B, Ratoff J, Meng Q, Mallett K, Cousins D (2005). Thymic stromal lymphopoietin expression is increased in asthmatic airways and correlates with expression of Th2-attracting chemokines and disease severity. J Immunol.

[b28-ehp-118-472] Zhou B, Comeau MR, De Smedt T, Liggitt HD, Dahl ME, Lewis DB (2005). Thymic stromal lymphopoietin as a key initiator of allergic airway inflammation in mice. Nat Immunol.

